# Cases in Practice

**Published:** 1876-04

**Authors:** A. R. Kilpatrick

**Affiliations:** Navasota, Texas


### CASES IN PRACTICE.

By A. R. KILPATRICK, M. D., Navasota, Texas.

Speedy Termination of Tedious Labor by Venesection.—Last May, 1875, a colored woman consulted me about her condition. She is below medium size, aged nearly forty, and the mother of three children then. Her general health good. About fourteen years ago her last child was born, who is now a stout boy. Soon after his birth, her husband quit her, and she formed an alliance with the man she now lives with. Her catamenia have been regular, but she has not been pregnant till now, and she is not certain whether she is or not, but the menses have "missed" for three times, and other concurrent symptoms induce me to believe she is pregnant, so I declined to give emmenagogues, but wait for further developments. She said she did not want any more children as her labors were all very tedious, and she was afraid, now, she would suffer more, or maybe die in the accouchment.

Some months after that, her husband reported to me that she was pregnant, and desired me to attend her when confined, if she needed any aid more than could be given by a colored midwife.

On the night of the 21st, November, I was called to see her, and reached the place between one and two o'clock, A.M. There was an elderly negro woman waiting on her, the mother of several children, not a professed midwife, but quite intelligent; also, a younger woman, very quick and sprightly, the mother of three children, and the wife of the patient's former husband.

The patient was at full term, the labor had begun three days before, on the 19th; the pains were slight and short, irregular in their recurrence, making the labor very slow indeed. She and both her attendants said no water had come from her at any time, and I observed that her clothes and bed were quite free from moisture. She frequently passed urine in the pot, which was, at times, streaked with blood, and there were red spots on her clothes, which I ascertained afterwards, came from the urethra. On examination, there was not the least sign of any membranes or amniotic fluid, although the vagina was sufficiently moist. She was very quiet; made very little noise or complaint, even when the pains were present, and had not slept, she said, since the labor began.

The presentation and position were the first of Bandelocque; the os tincæ was dilated, but thin; and when a pain was on, there was a tightening and rigidity. The upper portion was down like a hood over the head of the fœtus, holding it back. I tried, repeatedly, in the progress of the labor, to elevate and push back the hood, but did not succeed. She said the same trouble attended her other labors. The child's head was at the inferior strait of the pelvis, and remained there till a very short while before delivery.

I gave an enema and cleaned out the rectum, and ordered more black pepper tea, as she had been drinking it before. She had been standing and walking for the purpose of facilitating the progress of the labor, and I requested her to continue it as much as she could without fatigue.

There was nausea and occasional vomiting, which deceived me with the flattering hope that the rigidity and tension of the os uteri would soon subside, and the labor be terminated.

I folded a sheet diagonally and wrapped it as closely and tightly around her as she could bear, and made additional manual pressure during each pain. This caused an increase of the nausea, and she vomited a large quantity of bile, the pressure apparently producing duodenal regurgitation, and probably pressed out the bile from the gall-bladder. She complained of the pressure, the dyspnea and giddiness so much that I removed the bandage.

Finding the labor advanced so slowly, and wishing to end it as soon as possible and relieve her, I sent for some Fluid Extract of Ergot and my forceps, at 9 o'clock A.M. of the 22d. About 11 o'clock A.M. I gave some Ergot, and repeated it in half an hour, but instead of increasing the pains it caused a complete cessation. I then gave whisky with no better effect. During this lull in the labor she took a brief nap of sleep, the only time, she says, during the labor. I had employed Ergot in cases of threatened abortion, to stop it, but had no desire or expectation of its having that effect on this occasion.

I then procured the bark from living cotton plant roots, the *gossypium herbaceum*, which was cultivated around the house, and made strong tea and give it in two-ounce doses every half hour, but it produced no effect. After the Ergot was eliminated and its effects passed off, the pains returned, but still very mild, short and irregular.

As her strength continued good, and as the child's head was too high to apply the forceps, and she objected to their being used, fearing they would injure herself or the child, I concluded to try venesection; so I bled from the arm; the veins were small, and one failed to give blood enough to produce the desired effect, and another vein in the same arm was opened, from both of which over a quart of blood was drawn without producing syncope, but I feared to risk the loss of more blood, as there was some nausea, and she is of small frame, and had fasted quite three days.

It was nearly sunset. I had been with her over fifteen hours, and still there was very little change in the position of the fœtus.

Soon after the bleeding she wanted to go out in the yard, and I suppose she felt like having a stool; one of the women went with her, but she soon returned without having any evacuation. She looked wild, and gazed about foolishly, as though she was partially demented. I made her lay on the bed, and she still looked around foolishly. In less than half an hour after being bled, she told me she felt something like her womb coming out of her, yet she had not grunted, or made any complaint. I felt and found the child's head born and hanging between her thighs, and, with a little effort, the child was soon delivered—a male—sound and fully developed, though only weighing seven pounds. No liquor amnii was discharged at the time of birth. The woman did well and made a good recovery, after being in labor about four days and three nights.

The points of particular interest in this case are the failure of oxytocic effect in the remedies employed, for I cannot believe the gossypium tea had any, as there was no increase of pain, and no outcry at the supreme moment of the passage of the child's head. The ergot and whisky had the contrary effect, really stopping the pains, so that she enjoyed a slight, brief sleep at the time she was under their influence. The fact that she was delivered in so short a period after the bleeding, up to which time the child continued in very nearly the same position it was in fifteen hours before, satisfies me that the bleeding accomplished everything.

Her singular partially delirious conduct in the last stage of labor showed that the child's head was passing out, and my impression is, that if she had not been bled she would have had eclampsia at the time, and subsequently, which might have been attended with serious, if not fatal, results.

In conclusion, I would recommend the more general resort to the employment of the lancet in labor, tedious labors, especially in primiparæ.

Venesection lessens the dangers of eclampsia, relaxes the rigid os and cervix, the perineum and genitalia; and all these difficulties are usually encountered in persons of vigorous health, firm fibre and redundant flesh, which retard the egress of the child. Such persons bear the loss of blood beneficially.

Many, or most physicians and accoucheurs, now have a fear of venesection, lest the abstraction of blood should debilitate and prostrate the woman. There has long been a popular prejudice against the use of the lancet, and it is the duty of practitioners to endeavor to overcome and remove this prejudice, by well-timed argument and the judicious employment of venesection. The lancet was freely used for thousands of years, and the population of the world was not diminished by it. It was only the clamor raised against it by the notorious Samuel Thompson, about forty or fifty years ago, and kept up by his ignorant followers, conjoined with the timidity of regular practitioners, that brought it into discredit and almost disuse.

Of late years, since the practice of venesection has been almost abandoned, there is evidently an increase in the number of cases of puerperal fever, puerperal eclampsia, puerperal mania, phlegmasia doles, as well as an increased number of uterine disorders. I think this will be conceded by most of the old practitioners, and those who pay attention to the medical statistics. I candidly believe many of those cases of disease would not exist, or would be more easily remedied, if venesection was more often employed.

The entire absence of liquor amnii in this case is of importance. Dr. F. A. Barrall, of New York, reports a case in the October number, 1875, of the *American Journal of Medical Sciences*, page 446; and Dr. F. D. Lente, of Palatka, Fla., reports another in the same journal, in the January number, 1876, page 125. In commenting on this, he says some other physicians said they had met with such cases, while others regarded it as novel and interesting.

The practitioner is liable to be misled if there is a full caput succedaneum, and might scratch the scalp with the finger nail, or puncture it with an instrument. It is recommended to feel carefully for the hairs of the scalp before resorting to such measures.

